# A unique therapeutic approach to emesis and itch with a proanthocyanidin-rich genonutrient

**DOI:** 10.1186/1479-5876-6-3

**Published:** 2008-01-18

**Authors:** Mark JS Miller, Brian K Reuter, John L Wallace, Keith A Sharkey

**Affiliations:** 1 Center for Cardiovascular Sciences, Albany Medical College, Albany, New York, USA; 2 Department of Medicine, University of Virginia, Charlottesville, Virginia, USA; 3 Inflammation Research Network, Department of Pharmacology and Experimental Therapeutics, University of Calgary, Alberta, Canada; 4 Department of Physiology and Biophysics, University of Calgary, Calgary, Alberta, Canada

## Abstract

**Background:**

We examined the therapeutic potential of a proprietary *Croton palanostigma *extract (Zangrado^®^) in the management of emesis and itch.

**Methods:**

Emesis was induced in ferrets with morphine-6-glucuronide (0.05 mg/kg sc) in the presence of Zangrado (3 mg/kg, ip) and the cannabinoid receptor 1 antagonist, AM 251 (5 mg/kg, ip). Topical Zangrado (1%) was assessed for anti-pruretic actions in the 5-HT-induced scratching model in rats and evaluated in capsaicin-induced gastric hyperemia as measured by laser doppler flow. In the *Apc*^*Min*^mouse model of precancerous adenomatosis polyposis, mice received Zangrado (100 μg/ml in drinking water) from the age of 6 – 16 weeks for effects on polyp number. In RAW 264.7 cells Zangrado was examined for effects on lipopolysaccharide-induced nitrite production.

**Results:**

Zangrado was a highly effective anti-emetic, reducing morphine-induced vomiting and retching by 77%. These benefits were not associated with sedation or hypothermia and were not reversed by cannabinoid receptor antagonism. Itch responses were blocked in both the morphine and 5-HT models. Zangrado did not exacerbate the *Apc*^*Min*^condition rather health was improved. Capsaicin-induced hyperemia was blocked by Zangrado, which also attenuated the production of nitric oxide by activated macrophages.

**Conclusion:**

Zangrado is an effective anti-emetic and anti-itch therapy that is devoid of common side-effects, cannabinoid-independent and broadly suppresses sensory afferent nerve activation. This complementary medicine represents a promising new approach to the management of nausea, itch and irritable bowel syndrome.

## Introduction

The latex of the Amazonian traditional medicine *Croton palanostigma *and related *Croton *species is traditionally used in the treatment of inflammation, pain, itch, and a number of gastrointestinal afflictions that are common in the rainforest [1]. This traditional medicine is derived from a fast growing tree that is known by different names in various countries: in Peru it is called sangre de grado and in Ecuador, sangre de drago. We have found substantial scientific support for a number of these ethnomedical applications [2-4]. A central component of these benefits centers on its ability to suppress the activation of primary afferent nerves, supplemented by transcription-based and direct anti-inflammatory mechanisms of action [2-4]. The present investigation is focused on the utility of Zangrado^®^, an extract of *Croton palanostigma*, in treatment of emesis and itch.

While nausea and vomiting can severely affect the quality of life, there have been few therapeutic advances in its management in recent years and these conditions remain therapeutic challenges [5-10]. Itch may be considered as a cutaneous equivalent of nausea in terms of the involvement of primary afferent nerves, and is similarly lacking in new treatments [11]. The current pharmaceutical options in the treatment of nausea and vomiting are invariably associated with side-effects reflecting their actions on the central nervous system [5-7]. Perhaps not surprisingly given the restricted therapeutic options and high incidence of complications there is a high use of complementary medicines in this patient population [5]. An effective approach that is devoid of central nervous system complications remains elusive.

Cannabinoids have been a recent focus of potential therapeutic breakthroughs [8,12,13]. Cannabinoids are effective anti-emetics and analgesic agents. However, central nervous system complications, including sedation, hypothermia and other cognitive complications, remain a challenge [14]. In addition, the societal and legal issues that surround cannabinoids are not easy to overcome. The present investigation was designed to address the potential anti-emetic benefits of Zangrado by examining its actions in the presence of cannabinoid receptor antagonism, and by exploring its actions on various agents that activate primary afferent nerves.

## Methods

### Production and standardization of Zangrado

Zangrado was produced from the latex of the Peruvian medicinal plant, sangre de grado, by Rainforest Nutritionals, Inc. (Raleigh, NC). This proprietary technique yields an extract that is standardized for proanthocyanidin content (minimum of 100 mg/g) that is comprised of short-chain oligomers, less than 6 mer, as recently described [4].

### Opioid-Induced emesis in ferrets

Adult ferrets (900–1500 g, *Mustela putoris furo*, Marshall Research Labs, NY) were used and all experiments were conducted in accordance with the guidelines established by the Canadian Council on Animal Care and were approved by the University of Calgary Health Sciences Animal Care Committee. Animals were fasted overnight before experimentation.

Two series of experiments were performed. One focused on morphine-6-glucuronide (M6G) induced emesis, defined as episodes of vomiting and retching, and the other protocol compared the effects of Zangrado with WIN 55,212-2, a cannabinoid receptor 1 (CB_1_) agonist, on body temperature and sedation.

Zangrado (3 mg/kg, ip), the CB_1 _receptor antagonist, AM 251 (5 mg/kg, ip, Tocris, Ballwin, MO) or vehicle (2% dimethyl sulfoxide and 1% Tween 80 in physiological saline), were administered 15 minutes prior to the emetic agent M6G (0.05 mg/kg, sc, Lipomed, Arlesheim, Switzerland). Emesis was then measured for the next 60 minutes. The majority of emetic episodes occur within the first 10 minutes after M6G administration and the 60 min protocol duration is sufficient to capture all emetic episodes. Itch, like nausea and vomiting, is a common side-effect of opioid narcotics. In ferrets M6G treatment evokes a licking response with a similar time-course to vomiting and retching. It is not clear if this response reflects a response related to vomiting e.g., nausea, or rather does it mimic itch. Both sensations are difficult to model. While there is uncertainty as to what the licking response reflects it was nevertheless quantified.

In a separate series of experiments, the CB_1 _receptor agonist, WIN 55,212-2 (Tocris, Ballwin, MO) or Zangrado was administered to ferrets in the absence of M6G to quantify their effects on body temperature and ambulation. Hypothermia and sedation are common effects of cannabinoids. The dose of WIN 55,212-2 (1 mg/kg, ip) had previously been shown to suppress the emetic effects of M6G, an effect which is blocked by AM 251 at the dose used in the current M6G protocol (Van Sickle et al 2001, Simoneou et al 2001). Body temperature was measured rectally at 30 and 45 minutes after administration of WIN 55,212-2 or Zangrado. Ambulation was not quantified, but observational notes were taken.

### Scratching evoked by intradermal serotonin

As with the ferret studies, rat protocols were conducted in accordance with the guidelines established by the Canadian Council on Animal Care and were approved by the University of Calgary Health Sciences Animal Care Committee. Adult male Sprague Dawley rats who had previously had the fur clipped and shaved from the nape of the neck, received a subcutaneous injection of serotonin (5-HT, 100 μg) to evoke scratching behavior [15], with saline injections used as the sham control. Serotonin-evoked scratching is regarded as a reliable model of itch [16,17], in contrast histamine does not evoke this behavior in rats [16-18].

A time course response where the number of scratching episodes following subcutaneous 5-HT (2%, 10 μl) was determined over the course of a 1 hour observation period as previously described [15]. This was followed by two investigational protocols. In the first protocol 50 μl of a 1% Zangrado crème in a base of Dermabase (Glaxo) was applied either 30 min prior to the administration of 5-HT, or immediately after 5-HT administration. In the second protocol topicals were applied after immediately after the 5-HT injection, and a 1% Zangrado crème was compared to a commercial hemorrhoid crème (Preparation H^®^, American Home Products) or vehicle alone. Topicals (50 μl) was gently rubbed into the skin at the 5-HT injection site. Rats were then placed in individual cages to facilitate video-recording of behavior (in the absence of laboratory personnel) for 60 minutes. The frequency of scratching episodes was quantified by an individual unaware of the treatments the rats had received as previously described [15].

### Capsaicin-induced gastric hyperemia

As previously described [3], gastric blood flow was measured by a laser Doppler flow meter placed on the gastric luminal surface of anesthetized, laparotomized rats. This involves the use of a plexiglas chamber used for isolating the mucosal surface while maintaining the neural and vascular supply intact. The luminal surface is continually bathed in a buffered salt solution. After a 15 min baseline recording of blood flow capsaicin (320 μM) alone, or containing Zangrado (0.2 mg/ml) was added. Hyperemia is expressed as a percentage change from baseline.

### Murine familial adenomatosus polyposis (FAP) as a model of colon cancer

*Min*/+ (C57BL/6J-*Apc*^*Min*^) and wild type (C57BL/6J) mice were purchased from Jackson Laboratories (Bar Harbor, ME). Mice were housed in a room with a 12 hour light-dark cycle and had free access to food (LabDiet^®^, Prolab^® ^RMH 3500, Purina Mills, Inc., St. Louis, MO) and water during the entire protocol. All procedures were approved by the Albany Medical College Institutional Animal Care and Use Committee (IACUC).

*Min/*+ mice were treated for a period of 10 weeks either with vehicle (water) or Zangrado in drinking water at a concentration of 100 μg/ml. This concentration was chosen because a previous study showed that the parent traditional medicine, the latex of *Croton palanostigma*, effectively healed gastric ulcers in rodents via this route of administration at 20 or 200 μg/ml [2], without affecting behavior or drinking habits. Fluid intake and body weights were recorded every three days, with fluid intake calculated based on the number of mice per cage.

Treatment commenced at 6 weeks of age and concluded at 16 weeks of age. Following completion of the treatment period, mice were euthanized with sodium pentobarbital (Nembutal^®^, 50 mg/kg im.) and the entire gastrointestinal (GI) tract removed. The gastrointestinal tract was segregated into stomach, small intestine, and colon, and placed into 10% phosphate buffered formalin for fixation.

Following a 24 hour fixation period, the stomach and intestines were opened longitudinally and rinsed with phosphate buffered saline (PBS, pH 7.4). Tissues were pinned flat on wax blocks, covered with trypan blue (0.4% solution) for 10 minutes and rinsed with PBS to improve polyp contrast. Polyp numbers were determined by manually counting via a dissecting microscope (Bausch and Lomb, Rochester, NY) by an observer unaware of the treatment protocol for that preparation.

### LPS-Induced nitrite production

Lipopolysaccharide (LPS) induction of nitric oxide production by macrophages, reflecting the up-regulation of inducible nitric oxide gene expression, was quantified as nitrite accumulation [19]. Cells (2.5 × 105) were placed into 24 well plates and allowed to adhere overnight. Following the overnight incubation, the old media was aspirated and new media containing the test compounds and LPS (E. coli serotype 026:B6) were added (final volume per well equal to 1 ml). Cells were pre-incubated with the test compounds for 30 minutes, followed by a 24 hour exposure to LPS (2 ng/ml). Nitrite concentration was determined from an aliquot of the supernatant. Supernatant solutions were mixed with an equal volume of Griess reagent (1% sulfanilamide, 0.1 % napthylethylene diamine dihydrochloride, 5% phosphoric acid) and nitrite content measured spectrophotometrically at a 540 nm (reference wavelength 650 nm). Nitrite concentration was then extrapolated from a standard curve generated with sodium nitrite. Cell viability was determined via the MTT assay.

### Cell and culture conditions

Murine macrophages (Raw 264.7) cells were maintained in Dulbecco's modified Eagle medium (DMEM) with fetal bovine serum (10%), 4.5 g/L D-glucose, 4 mM L-glutamine, 25 mM HEPES and 1.5 g/L sodium bicarbonate. Culture medium contained 40 μg/ml penicillin G, 40 μg/ml streptomycin and 0.1 μg/ml amphotericin B. Culture media was replaced every three days and cell lines were subcultured weekly with trypsin. Cells were incubated in a humidified chamber at an atmosphere of 5% CO_2 _and 37°C. Culture reagents were purchased from Mediatech, Inc (Herndon, VA).

### Statistical evaluation

Data is presented as mean ± SEM with annotation as to the n value. Comparisons between groups for simple paired comparisons were made with a Students' t-test. However, if there was more than one group the data was initially examined by ANOVA, and if significant then an appropriate multiple comparison test was used (Dunnett's or Bonferroni). Significance was taken at a p value of less than 0.05.

## Results

### Emesis and Itch induced by morphine-6-glucuronide

**Figure 1:**
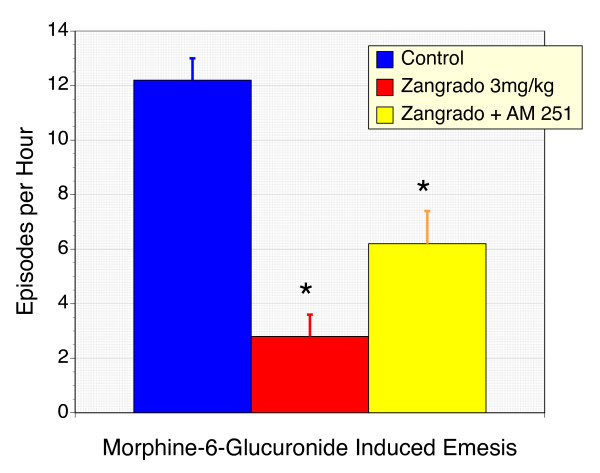
**Suppression of morphine-6-glucuronide-induced vomiting and retching in ferrets with Zangrado, and the effects of CB_1 _receptor antagonism with AM 251**. Ferrets treated with the opioid narcotic, morphine-6-glucuronide (M6G) display a substantial emetic response with on average over 12 episodes per hour. Zangrado administration (3 mg/kg ip) 15 minutes prior to M6G resulted in a 77% reduction in emetic episodes (n = 6, P < 0.01). Co-administration of Zangrado with the CB receptor antagonist, AM 251 (5 mg/kg, ip) failed to reverse the anti-emetic effects of Zangrado (n = 6). The slight increase in emetic episodes with AM 251 is comparable to that seen with ferrets treated with AM 251 alone and is thought to reflect the anti-emetic effects of endogenous cannabinoids [20,21].

**Figure 2:**
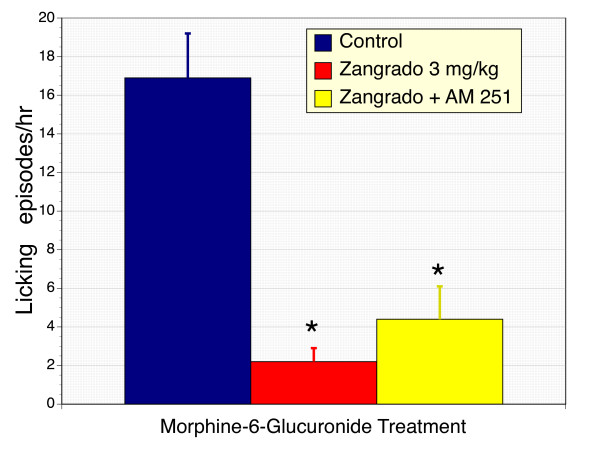
**Suppression of morphine-6-glucuronide-induced licking in ferrets with Zangrado, and the effects of CB_1 _receptor inhibition of AM 251**. Ferrets treated with M6G display a licking response which was quantified. Treatment with Zangrado (3 mg/kg, ip) reduced this licking response by 87% (n = 6, P < 0.01). Co-treatment with AM 251, the CB_1 _receptor antagonist, failed to reverse the benefits of Zangrado on M6G-induced itch (n = 6).

Ferrets respond to M6G with a rapid and reproducible emetic response (vomiting and retching) that typically abates within 10 minutes. Previously, we have shown that this response is suppressed by CB_1 _receptor agonism and enhanced by CB_1 _receptor antagonism [20,21]. At the dose of 3 mg/kg, Zangrado was a remarkably effective anti-emetic reducing episodes of vomiting and retching in M6G treated ferrets by 77% (Figure 1). Co-administration of the CB_1 _receptor antagonist, AM 251, failed to reverse this response, although the number of episodes did increase. However, the magnitude of this AM 251 effect was comparable to what we have previously observed in the M6G model with AM 251 in the absence of Zangrado. In other words, AM 251 significantly raises emetic episodes in M6G treated ferrets because it blocks the anti-emetic tone exerted of endogenous cannabinoids [20]. The number of licking episodes, was reduced by 87% with Zangrado (Fig. 2). This effect was not reversed by AM 251.

**Figure 3:**
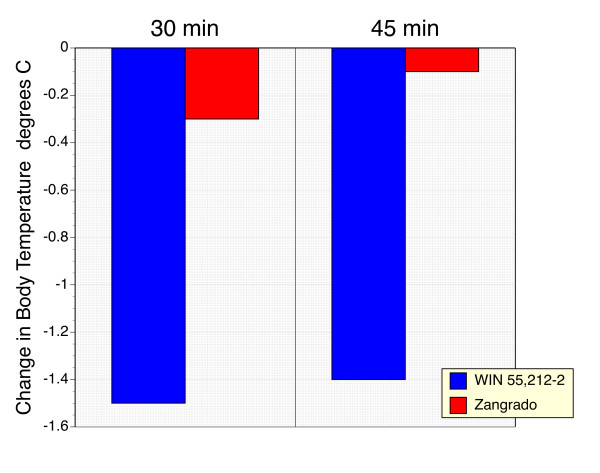
**Comparison of the cannabinoid receptor agonist, WIN 55,212-2 and Zangrado on body temperature in ferrets**. Ferrets treated with the CB_1 _receptor agonist, WIN 55,212-2 (1 mg/kg, ip) displayed a substantial reduction in body temperature when measured at 30 and 45 minutes after administration. In contrast, there was no effect of Zangrado (3 mg/kg, ip) on body temperature (n = 6, p < 0.05). Both agents were administered at effective anti-emetic doses.

To further compare the anti-emetic actions of Zangrado with cannabinoids and to evaluate the potential side-effects of Zangrado, we compared Zangrado and the CB_1 _receptor agonist WIN 55,212-2 on ferret body temperature and motility. Using a dose of WIN 55,212-2 previously shown to suppress M6G-induced emesis [20-22], we noted a profound and sustained hypothermic response (Figure 3). However, in Zangrado treated ferrets body temperature remained unaltered. Ambulation was not directly quantified as an index of sedation, but subjective measurements were clear-cut. Ferrets treated with WIN 55-212-2 lay essentially motionless in their cages during the observation period. In contrast, Zangrado treated ferrets displayed a normal playful movements and freely explored their environment and performed grooming behaviors.

### Scratching evoked by subcutaneous 5-HT

**Figure 4:**
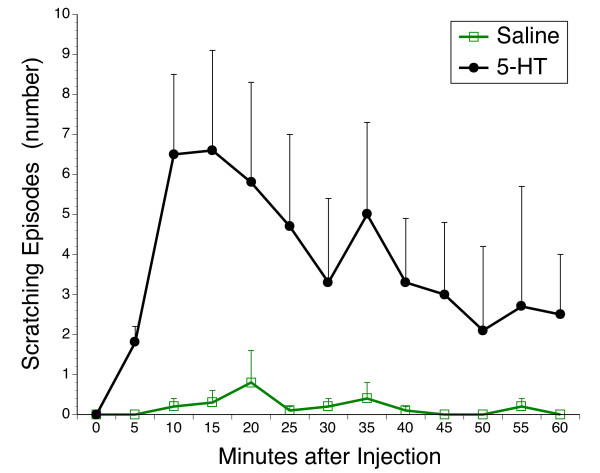
**Time course of the scratching response to subcutaneous 5-HT**. Number of episodes of scratching in 5-min intervals over a one-hour period after injection of 5HT subcutaneously. (n = 5 per group) Controls got an injection of an identical volume of saline (100 μl).

Local administration of 5-HT evokes a scratching response at the site of the injection that peaks within 10 minutes and then slowly declines over the course of the 60 minute observation period (Figure 4). By contrast saline did not evoke a scratching response.

**Figure 5:**
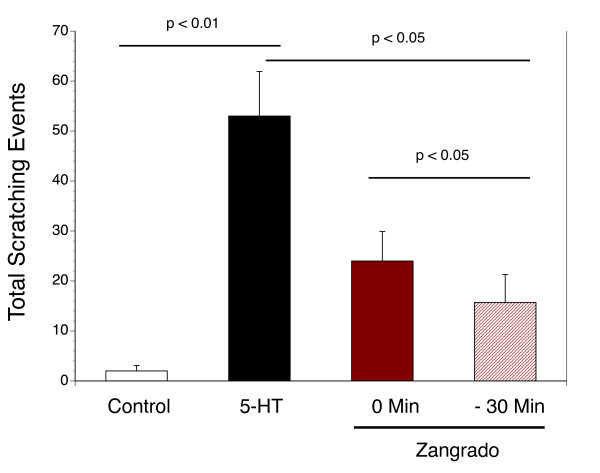
**Effects of Zangrado on 5-HT evoked scratching: Comparison between pretreatment and immediate post-treatment**. Scratching episodes in response to saline injection (control, n = 5) and 5-HT (black column, n = 11) with vehicle treatment (50 μl of Dermabase crème) or the effects of 50 μl of zangrado (1%) crème applied 30 min (-30 Min column, n = 10) before the sc injection of 5-HT or immediately after (0 Min column, n = 8). The statistical difference between groups is noted in the figure.

**Figure 6:**
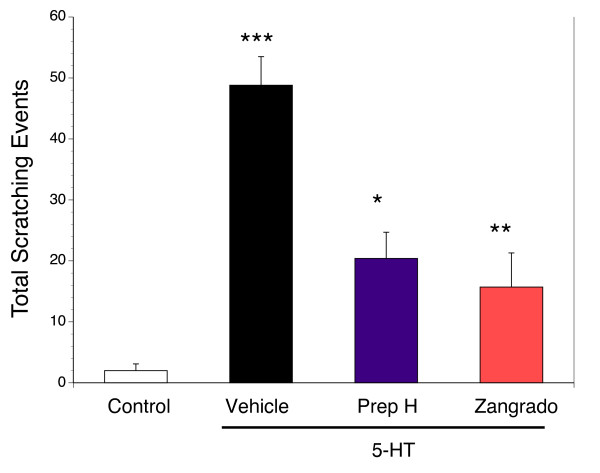
**Anti-pruretic effects between Zangrado and a commercial hemorrhoid crème**. Subcutaneous 5-HT administration (black column, n = 11) evoked a significant scratching response over saline (control, *** p < 0.001). This scratching response was significantly attenuated by either topical administration of Preparation H (Prep H, purple column, * p < 0.01, n = 7) or Zangrado (red column, ** p < 0.001, n = 10), when applied immediately after the administration of 5-HT.

Topical administration of Zangrado (1%) was effective in suppressing the number of scratching episodes induced by 5-HT (Figure 5). There was a significantly greater reduction in the scratching response when Zangrado was administered 30 minutes prior to 5-HT administration versus immediately after 5-HT (p < 0.05), but both were effective anti-pruretic regimens (p < 0.05) compared to vehicle. When evaluated against a commercially available hemorrhoid crème (Preparation H) as an anti-pruretic therapy, both agents significantly reduced the total number of scratching episodes (Figure 6) with the Zangrado being slightly more effective although this difference was not statistically significant.

### Gastric hyperemia induced by topical capsaicin

**Figure 7:**
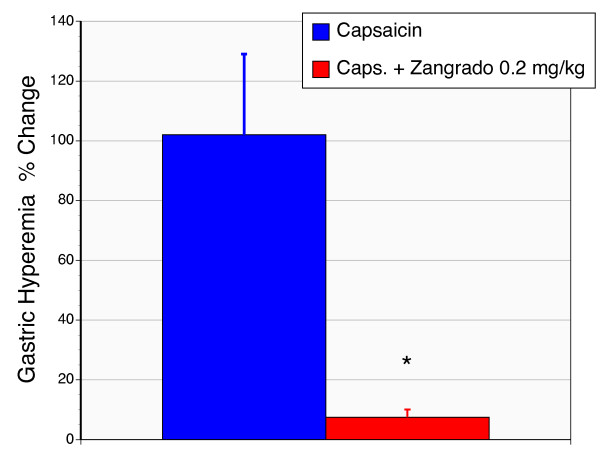
**Effect of Zangrado on capsaicin-induced gastric hyperemia in rats**. Capsaicin (320 μM) applied to the luminal surface of intact gastric mucosa results in a hyperemia as measured by laser Doppler flow. Rats pretreated with luminal Zangrado (0.2 mg/kg) ablated this hyperemia response (n = 5, P < 0.01).

Capsaicin applied topically to the gastric mucosal surface resulted in a marked increase in local blood flow (Fig. 7). Zangrado administered as a pretreatment (0.2 mg/kg) was able to completely prevent this hyperemia response.

### Effects of chronic Zangrado in Apc^*Min/+*^mice

**Figure 8:**
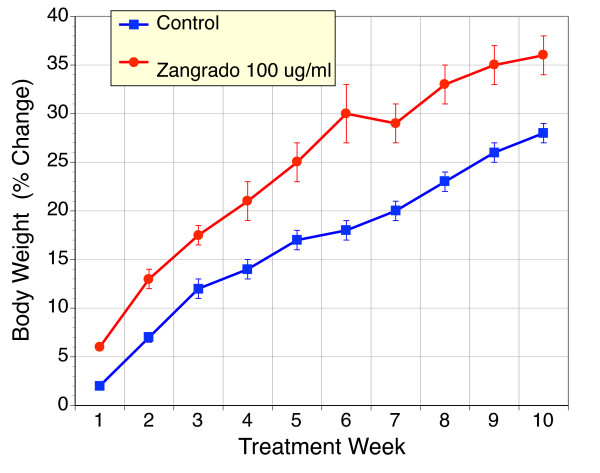
**Body Weight in *Apc*^*Min/+*^mice over a 10 week treatment period with Zangrado or vehicle**. Body weight (mean ± sem) of *Apc*^*Min/+*^mice determined over a 10 week treatment period is shown for mice treatment with Zangrado (n = 5, 100 μg/ml in drinking water) or unaltered drinking water (control). The Zangrado treated mice displayed a greater body weight over this period than did control mice (n = 10). This trend was not statistically significant.

**Figure 9:**
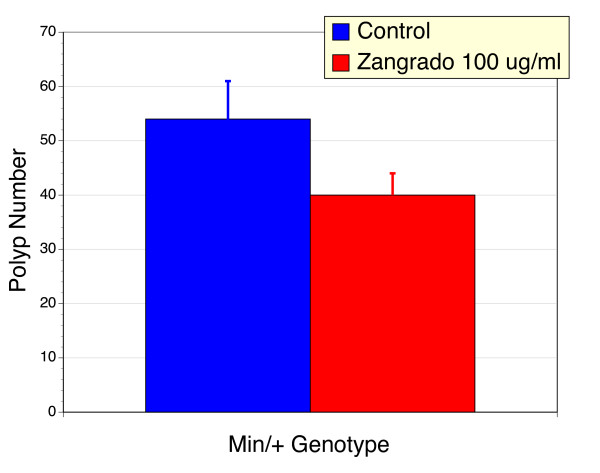
**Polyp number in control or Zangrado treated *Apc*^*Min/+*^mice**. *Apc*^*Min/+*^mice treatment with Zangrado (100 μg/ml in drinking water, n = 5) displayed a 26% reduction in the number of polyps when compared to vehicle control mice (n = 10). This trend was not statistically significant. There were no polyps noted in wild type mice.

The *Apc*^*Min/+*^murine model of FAP and colon cancer is characterized by the development of a large number of polyps in the small and large intestine [23]. Given that Zangrado may be potentially used to control nausea and emesis in cancer patients, we elected to determine the impact that Zangrado may have on polyp development in this model. Weight gain in *Apc*^*Min/+*^mice is compromised as a result of their condition. When Zangrado was added to the drinking water (100 μg/ml) for 10 weeks beginning at 6 weeks of age, there was a tendency for an improvement in weight gain (Figure 8). At necropsy the number of polyps in Zangrado treated *Apc*^*Min/+*^mice was reduced compared to control mice (Figure 9). Both the changes in weight gain and polyp number failed to reach statistical significance, but this may reflect the small n values in this evaluation for a model with inherent variability.

### LPS-induced nitrite production by macrophages

**Figure 10:**
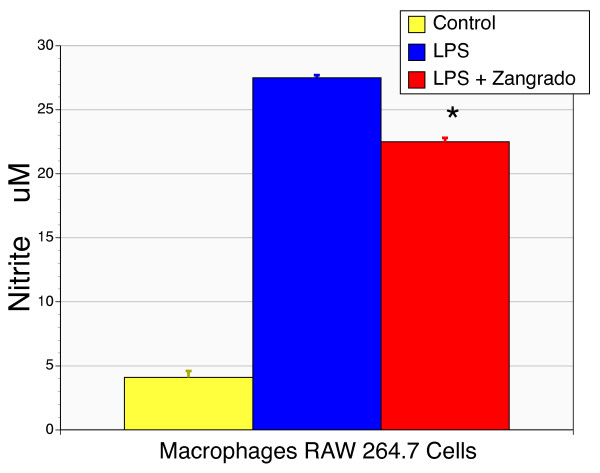
**Effects of Zangrado on the production of nitrite by LPS-activated macrophages**. Murine macrophages, Raw 264.7 cells, treated with LPS (2 ng/ml) displayed a marked increase in nitrite production. Co-administration of Zangrado with LPS resulted in a significant reduction in nitrite production (n = 6, p < 0.05).

Murine macrophages treated with LPS accumulated nitrite in the media as previously described, reflecting the induction of inducible nitric oxide synthase gene expression [19]. Treatment with Zangrado resulted in a suppression of nitrite levels (p < 0.05, Figure 10)

## Discussion

These results indicate that Zangrado, a proprietary extract of the Amazonian medicinal plant *Croton palanostigma *[4] is an effective anti-emetic agent in the opioid-induced model in ferrets. Indeed, in this regard it is remarkably potent given that it is a natural product and not pure single chemical entity and administered at 3 mg/kg. The magnitude of this anti-emetic action is substantial, even when compared to pure cannabinoid agonists [20,21] administered at comparable doses (1 mg/kg). Beyond efficacy it is indeed important to note that severe sedation that accompanies cannabinoid-based anti-emetic actions were completely absent with Zangrado.

This opioid narcotic model of emesis is also associated with licking response, which may reflect itch, another characteristic complication of post-operative narcotic analgesia. Zangrado was similarly effective in blocking both emesis and licking in response to morphine. Both itch and nausea/emesis are mediated by primary afferent nerves, and morphine is thought to promote both sensations secondary to spinal disinhibition of suppressive of pain pathways [11]. These are troubling complications of post-operative narcotic analgesia [9] and the present results suggest that Zangrado may represent a new approach to improved patient care.

Cannabinoids can also attenuate both itch, emesis and nausea [12-14,20-22] and given the phytoceutical nature of cannabinoids and Zangrado it was deemed important to evaluate if the effects of Zangrado were mediated by cannabinoid receptors. However, AM 251, the CB_1 _receptor antagonist, failed to block the anti-emetic or anti-licking effects of Zangrado in the M6G model were blocked (Figures 1 and 2). We have previously reported that this dose of AM 251 is effective in preventing the anti-emetic actions of the cannabinoid agonist WIN 55,212-2 [20]. From this we conclude that Zangrado's anti-emetic actions are not mediated by CB_1 _receptors. The slight increase in emetic and licking episodes with AM 251 is consistent with an action on endogenous cannabinoids and independent of Zangrado's actions.

Additionally treatment of naïve ferrets with the CB_1 _receptor agonist WIN 55,212-2, administered at an effective anti-emetic dose [20-22], caused a series of significant and characteristic side-effects, specifically sedation and hypothermia (Figure 3). In contrast, Zangrado failed to alter body temperature, motility and behavior. These results set Zangrado apart from cannabinoid-based approaches.

Primary afferent nerves are responsible for transmitting the sensations of pain, itch and nausea, with the latter being organ-specific responses. Recently it has been demonstrated that primary afferent fibers that mediate the sensation of itch as opposed to pain [24,25], represent a separate subclass of these nerves. This finding was in keeping with the knowledge that opioid narcotics could block pain and spinal responses to capsaicin but yet evoke itch and nausea, as reproduced in this study [9]. Counter-irritancy has also been a therapeutic approach to itch to manage the dominance of one symptom but is ultimately a less than desirable approach to treating these conditions.

Previously we had demonstrated that *Croton palanostigma *latex was an effective analgesic and inhibitor of neurogenic inflammation [2,3]. This was extended to Zangrado in the present study where gastric hyperemia induced by mucosal capsaicin administration was blocked. It is well appreciated that capsaicin's hyperalgesia and vascular actions result from the activation of primary afferent nerves via the transient receptor potential vanilloid receptor 1 (TRPV1) and this action suggests that Zangrado has a local action to limit sensory afferent neural activity. Indeed, we had noted that *Croton palanostigma *blocked inflammatory events (hyperalgesia, edema, vasodilation) induced by a diversity of agonists for primary afferent nerves, including capsaicin, PGE_2 _and protease activated receptor-2, recently implicated in the pathophysiology of irritable bowel syndrome [26]. This suggests that primary afferent nerves are the likely target for this traditional medicine. This interpretation is extended in the present study where the itch response to both morphine and subcutaneous 5-HT were blocked despite their central versus local nervous system origins. It is uncommon for a therapeutic agent to block pain, itch and nausea, and strongly suggests that these benefits result from a broad suppression of primary afferent nerve activity and not an effect on a specific subset of nerves (emesis versus pain, or itch versus pain). Irritable bowel syndrome (IBS) is a major gastrointestinal disorder that remains poorly managed. Nevertheless as it is well accepted that a critical and driving component of the disease is an overly reactive primary afferent nervous innervation of the gut [26], the calming actions of Zangrado on this neural network may represent a potential therapeutic breakthrough that should be explored.

The lack of sedative effects or hypothermia associated with Zangrado's benefits further supports the concept that benefits are directed at the peripheral and not central nervous system. Generally, the current options for treating nausea and vomiting possess a central nervous system mechanism of action [8]. Agents that solely operate via peripheral actions are generally less effective (glucose, ginger). Further support for a peripheral site of action for Zangrado is evident in a small clinical study where the parent traditional medicine was applied topically to various insect bites, stings and plant reactions [3]. In this study analgesic, anti-pruretic and other anti-inflammatory benefits were apparent within minutes of topical administration (1% solution) when applied after the event, similar to the present rat model of pruritis evoked by 5-HT. The rapidity of these benefits on topical application suggests a local action and is consistent with the traditional use of the medicinal plant in the Amazon rainforest where such afflictions are commonplace [1]. The other traditional use is for severe gastrointestinal events – nausea, ulcers, cramping and diarrhea. In our previous studies with *Croton palanostigma*, we noted improved gastric ulcer healing, suppression of neurogenic intestinal electrolyte fluxes, analgesia, and anti-inflammatory actions consistent with the traditional applications for the parent medicinal plant [2,3]. Indeed, it appears that suppression of neurogenic inflammation is an important component of these actions.

There are results that suggest that the traditional medicine possesses additional actions, which is not unexpected given that this is not a single chemical entity. The accelerated gastric ulcer healing in response to *Croton palanostigma *was accompanied with lowered expression of various pro-inflammatory genes [2]. This included inducible nitric oxide synthase, which is supported with the lowered macrophage production of nitrite in the present study and consistent with a redox-based modification of transcriptional events. With its high proanthocyanidin content, Zangrado is an exceptional antioxidant. A related extract progrado, which is enriched for log chain proanthocyanidin oligomers as opposed to short chain oligomers in Zangrado, has a number of anti-inflammatory and reparative actions that reflect transcriptional actions [4]. Progrado was effective in protecting human cartilage from the destructive effects of interleukin-1β, while promoting the expression of the anabolic repair factor, IGF-1. In addition, progrado can completely inhibit the gelatinolytic actions of matrix metalloproteinase 2 and 9 [4].

Standardization of botanical extracts is always of concern, as there is a potential for mixed results with different samples reflecting production, geographical or seasonal influences. As the precise chemical entities responsible for these actions are unknown we need to design a standardization process that is meaningful and reliable. For this extract we choose proanthocyanidin content and nature, specifically a proanthocyanidin content of > 100 mg/g, which is substantial, and composed of short-chain oligomers [4]. It is important to note that we have no encountered any definable variation in bioactivity over region and time in our investigations over the course of some years. Complex chemical extracts that constitute complementary medicines like Zangrado and Progrado by virtue of their mixed composition can provide a plethora of actions. In theory these various components may (a) enhance therapeutic potential, (b) exert independent actions or (c) potentially cancel benefits. Nevertheless, the anti-emetic and anti-pruretic actions in the present report suggest that Zangrado is not only effective but possesses characteristics that offer advantages over current therapeutic options.

The *Apc*^*Min/+*^murine model of colon cancer [23] was chosen for evaluation because nausea and emesis are significant factors in the management of cancer, chemotherapy and palliative medicine in general [10]. In these states it is important that any therapy does not exacerbate the cancerous state while alleviating the symptoms, hence the evaluation in the *Min/+ *model of familial adenomatous polyposis. We have previously observed that the *Croton palanostigma *promotes apoptosis in a variety of gastrointestinal cancer cell culture lines [27] suggesting that it may be a source of new oncological therapies. However, it was important to take these in vitro observations into a more complex animal model. The *Apc*^*Min/+*^mice studies showed improvements in weight gain and polyp number, and although encouraging these results did not reach statistical significance. Nevertheless, it was clear that Zangrado did not exacerbate the condition. By comparison the 28% reduction in polyp number with Zangrado was more effective than our previous observations with aspirin [28], a proposed colon cancer chemopreventative agent [29], which was associated with a 3% increase in polyp number and no improvement in animal weight gain. Proanthocyanidins, polyphenols that are exceptionally enriched in Zangrado, have been suggested to be potential chemotherapeutic agents in various cell lines [30], indicating that this chemical class may contribute to the actions of Zangrado. It is therefore tantalizing to consider that Zangrado may be a source of chemotherapeutic agents or adjuvants that also block nausea and emesis as opposed to promoting these adverse conditions. Safety is always an issue with the application of traditional medicines from outside cultures. In the case of Zangrado, the data is consistent with safe applications, as noted here with the chronic administration in *Apc*^*Min/+*^mice, as well as previous studies using OECD safety and toxicity tests where it was awarded the highest safety rating with no detectable toxicity at 2000 mg/kg on acute oral administration [4]. This is framed by the impressive potency of Zangrado as an anti-emetic, anti-pruretic agent and its ability to suppress primary afferent nerve activation in the present study. These mechanism of action results are entirely consistent with the low amounts *Croton palanostigma *used ethnomedically by cultures in tropical South America to manage these symptoms. We therefore contend that these mechanisms are the most likely explanations for the benefits of this traditional medicine, and because these actions are distinct from current treatment options Zangrado may represent a new and exciting approach to these conditions.

## Conclusion

Zangrado is a proanthocyanidin rich complementary medicine that has potent anti-emetic and anti-itch actions that are independent of cannabinoid receptors and appear to reflect actions on the peripheral nervous system, specifically primary afferent nerves. The paucity of side-effects that burden other anti-emetics suggest that Zangrado may be a useful therapeutic option to improve the quality of life for a variety of afflictions and complications where afferent nerve traffic is problematic including nausea, itch, chemotherapy, motion sickness, irritable bowel syndrome and palliative medicine.

## Abbreviations

CB_1 _cannabinoid receptor 1

FAP familial adenomatosus polyposis

GI gastrointestinal

LPS lipopolysaccharide

M6G morphine-6-glucuronide

PGE_2 _prostaglandin E2

5-HT 5 hydroxytryptamine, serotonin

## Competing interests

MJSM is a scientific advisor to Rainforest Nutritionals, Inc., which supplied the Zangrado for these studies. For these services he has received an equity position but not other financial compensation. There are no competing interests with the other authors.

## Authors' contributions

MJSM assisted with experimental design, data review and manuscript preparation

BKR performed the cell culture and cancer studies and manuscript preparation

JLW contributed with the 5-HT and capsaicin experiments and manuscript preparation

KAS contributed with the emesis studies and manuscript preparation.

All authors have read and approved the final manuscript.
